# Polyimide-Derived Supramolecular Systems Containing Various Amounts of Azochromophore for Optical Storage Uses

**DOI:** 10.3390/polym15041056

**Published:** 2023-02-20

**Authors:** Andreea Irina Barzic, Ion Sava, Raluca Marinica Albu, Cristian Ursu, Gabriela Lisa, Iuliana Stoica

**Affiliations:** 1 “Petru Poni” Institute of Macromolecular Chemistry, 700487 Iasi, Romania; 2 Faculty of Chemical Engineering and Environmental Protection “Cristofor Simionescu”, “Gheorghe Asachi” Technical University of Iasi-Romania, 700050 Iasi, Romania

**Keywords:** flexible polyimide, azo derivative, photo-induced properties

## Abstract

The progress of digital technologies demands more speed and larger storage capacity. Optical storage systems have the advantage of being cheap, fast and capacious. This article explores the potential use of polyimide-based films as a recording medium for optical storage devices. The materials were designed through a host–guest approach that involves a cyano-containing polyimide precursor and an azochromophore combined in the following ratios: 1:0.25, 1:0.5, 1:0.75 and 1:1. After thermal treatment up to 200 °C, polyimide systems were formed with supramolecular structures constructed via hydrogen bonding as shown by molecular modeling and FTIR at around 3350 cm^−1^. The aspects arising from the variation of the azo-dye content in the polyimide samples and their impact on the vitrification temperature, colorimetric features, refractive index, band gap, non-linear optical susceptibility and birefringence were investigated for the first time. The thermal analysis indicated a slight decrease in the vitrification temperature from 190.84 °C for the sample without azo dye to 163.91 °C for the film containing the highest leading of azo dye. The morphology images revealed the occurrence of periodic structures in azo-derived materials exposed to a UV laser, which is accentuated by the addition of more azo dye molecules. Optical tests allowed observation of the increase in the dominant wavelength, refractivity and optical conductivity of the samples, produced by the incorporation of azochromophore and laser irradiation. The photo-generated birefringence increased from 0.014 (sample with 1:0.25) to 0.036 (sample with 1:1), which in combination with the created regular topography pattern, is essential for the use of these materials as recording media in optical storage applications.

## 1. Introduction

Polyimides (PIs) represent one of the most relevant kinds of thermally resistant polymers employed in a wide range of applications, such as aerospace, membranes, adhesives, automotive and in a variety of electronic devices [1,2,3]. For implementation in electronic products, the material must exhibit distinctive features, aside from thermal stability, such as flexibility, chemical resistance, dimensional stability, relatively low permittivity and a specific molecular arrangement [4]. The latter feature can be achieved as a function of chain conformation, which can be further tuned by the presence of certain external factors (radiations or mechanical forces) [5,6,7]. In their absence, the route for adaptation of the PI properties relies on controlling the polymer chemical structure by carefully selecting the combination of monomers used in synthesis, which, generally, are diamines or diisocyanates in reaction with dianhydrides [8]. Therefore, as a function of each monomer feature (i.e., size, flexibility, symmetry, level of conjugation, etc.), it is possible to attain the targeted conformational characteristics [9], with a deep impact on the balance of thermal, mechanical, optical and electrical properties [10,11]. For example, incorporation of cyano groups in the PI structure provides additional chain flexibility and opportunities for crosslinking while enhancing the thermal stability, and through its large polarization and low molecular volume, it augments the dielectric constant in comparison with the PI counterparts lacking this group [12].

Particular attention is given to photodeformable PIs owing to the noteworthy features generated by radiation that enable their spatial and directional control [13]. To achieve photosensitive PI films, it is preferable to insert photo-switchable chromophores into the material structure, which is an approach that allows the extension of the molecular scale motions up to the macroscopic level. Azo derivatives are the top choice photosensitizer due to their ability to undergo reversible trans−cis and cis−trans isomerization in the presence of ultraviolet (UV) and visible radiation [14,15]. The photo-induced properties are strongly affected by the characteristics of the incident radiation, such as the wavelength, polarization state and irradiation time, as well as the use of masks that allow selective irradiation of the polymer surface. The geometrical modification of the molecules attributed to the azo-compound isomerization may eventually lead to macroscopic deformation of polymeric materials, for example, PIs [16]. During laser exposure, the unidirectional orientation of the chromophore occurs as an outcome of the light-triggered isomerization among the configurations of the azo-based unit. When such a molecule gains a transition dipole aligned at 90° to the polarization vector, it determines a null transition dipole momentum. Hence, no supplementary excitations take place, and the material displays a change in the orientation of the optical axis with regard to the position of the radiation electric vector [17]. By modifying the polarization state of the incident radiation, the material can be commutated reversibly between two states of distinct molecular arrangement. For instance, an isotropic in-plane disposition of the polymer chains can be converted into a powerful anisotropic orientation via exposure to linearly polarized radiation [18]. In this way, the refractive index recorded along the polarization direction (named n_x_) tends to shrink in magnitude, whereas the in-plane index orthogonal to this direction (n_y_) is augmented. The original isotropic material might be optically restored when it is under the effect of circularly polarized radiation. The large photo-generated and erasable birefringence (Δn = n_y_ ± n_x_) values are of great scientific interest [18].

As a result of the chromophore rearrangement caused by light irradiation within the polymer system, the overall material gains new properties such as birefringence, dichroism and textured topography [19]. These characteristics could be exploited for the development of polymer-based sensors [20], photoactuation [21] and optical storage [22] devices. For the last application, polymers displaying optical sensitivity can be utilized as optical recording media. The writing procedure entails selective excitation of the azo-based moieties that suffer numerous trans–cis–trans isomerization accompanied by reorientation, hence augmenting the amount of photochromic groups orthogonal to the polarization direction. Such a mechanism implicates the movement of the side groups and the backbone under the polymer glass transition temperature (Tg) [23]. The stable birefringence pattern indicates good storage ability of the optical medium. The combination of bulk birefringence and the production of a surface relief pattern are advantageous for attaining recording layers that are increasing gain effect in holographic storage. In contrast to digital storage, the holographic one can be accomplished by registering the interference pattern of radiation that carries the optical data [24]. During the photoanisotropic modulation stage, the data are registered in the polymer sheet as dichroism and birefringence, whereas for the surface modulation, the data are registered as variations in the sample configuration.

Related to the preparation strategy of photo-sensitive PI materials based on azo derivatives, there are two general possibilities. The first one is concerned with utilization in polymer synthesis of monomers that contain azo-moiety in their structure [25], while the other method involves the addition of azo derivatives in the PI host [26]. An interesting approach to creating a supramolecular architecture of such materials is via hydrogen bonding connection between the chromophore and PI host, as described in the works of Schab-Balcerzak [27,28]. The reports are mainly focused on chromophores which comprise a pyridine ring as an alternative for phenyl of azobenzene or a matrix that includes pyridine units. As far as we know, the effect of the azochromophore amount on the PI conformational, thermal and optical properties before and after laser exposure has not been studied yet.

A previous article [29] focused on the photodesign of regular surface patterns in supramolecular systems made from a photosensitive PI and another azo dye. The host PI used in [29] is prepared from a carbonyl-containing dianhydride and a flexible diamine containing ether and methyl kinks (lacking the cyano group). The surface topography was evaluated at the nanoscale (before and after phase mask UV laser), revealing that a higher pattern regularity and surface anisotropy are optimal for orientation of the nematic molecules. A more recent work [30] had the goal of analyzing the effects induced by the type of azo dye and on the thermal degradation behavior, colorimetry parameters and morphological characteristics. Moreover, the cyano group from the diamine is placed in the ortho-position, and the outcome produced by mask UV laser treatment on these samples is investigated in relation to flexible electronic substrates use. In another work [31], a novel aromatic diamine containing cyano groups was prepared. This monomer led to soluble and flexible PIs, with raised thermal resistance. It was also shown that PIs containing cyano units in the diamine moiety display good mechanical properties and a very flat morphology [32].

Starting from these premises, this article is motivated to investigate, for the first time, certain original insights arising from the implications of azo-dye content in polyimide samples. The PI host structure used here contains the cyano group in the metha position and is combined with an azochromophore that has cyano as the terminal group. These new structural aspects are studied with regard to the possibility of adapting the thermal properties, colorimetric features and optical parameters (refractive index, band gap, non-linear optical susceptibility, optical conductivity and birefringence). Laser exposure of the examined films was carried out using the same kind of phase mask, and it is generating bulk orientation, which is reflected in a regular topography pattern and enhanced birefringence. Both features are indicative of proper writing of the information in the polymer recording medium, as imposed by the optical storage applications.

## 2. Materials and Methods

### 2.1. Materials

In this study, the following materials were used: 4,4′-isopropylidene-diphenoxy-bis(phthalic anhydride) (6HDA), which was purchased from Sigma-Aldrich, St. Louis, MO, USA; and 2,6-bis(3-aminophenoxy)benzonitrile (m-CN) and 4-[(4-cyanophenyl)diazenyl]phenol (AzoCN) synthesized in our laboratory using previously reported methods [33,34,35,36] at M.p. 136–138 and 200–202 °C, respectively. N,N-dimethylacetamide (HPLC grade), p-aminobenzonitrile, phenol (99%) and NaNO_2_ (99.9%) were purchased from Merck KGaA, Darmstadt, Germany. All reagents and solvents were used as received.

### 2.2. Polyamidic Acid (PAA) Synthesis

Using polycondensation reaction with equimolar quantities of 2,6-bis(3-aminophenoxy) benzonitrile (m-CN) and 4,4′-isopropylidene-diphenoxy-bis(phthalic anhydride) (6HDA), polyamidic acid was obtained. The reaction was undertaken in DMAc at a total concentration around 15%, at room temperature and under nitrogen atmosphere for 4 h. The obtained PAA solution was used in the next stage of realizing the supramolecular systems.

### 2.3. Polyimide-Based Supramolecular System

**Scheme 1:**
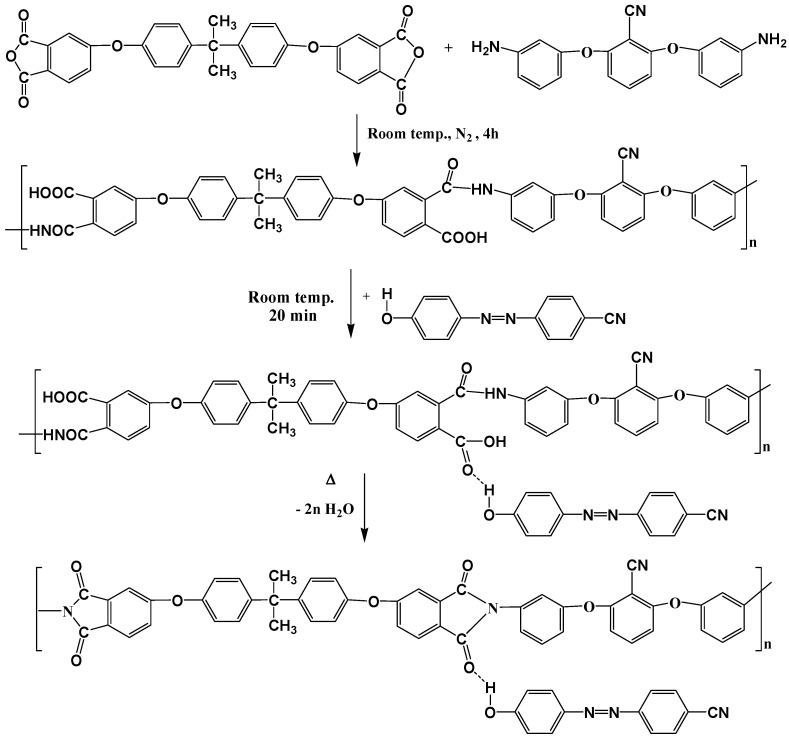
Step-by-step evolution of the reaction that generates the final PI-based supramolecular system.

To realize the supramolecular systems, in the solution of polyamidic acid was introduced the azochromophore 4-[(4-cyanophenyl)diazenyl]phenol (AzoCN) in different ratios, followed by casting onto glass plates and thermal treatment. Thus, the molar ratio between polyamidic acid and azomonomer was 1:1 (SPIN100), 1:0.75 (SPIN75), 1:0.5 (SPIN50) and 1:0.25 (SPIN25). The pristine PI film based on m-CN and 6HDA was abbreviated SPIN0. The used quantities are presented in Table 1. The step-by-step structural evolution of the reaction is depicted in Scheme 1. By casting guest–host PAA solutions on glass plate, followed by thermal treatment, flexible films with imide structures were obtained. The thermal treatment was realized starting at 50 °C for 4 h, followed by slowly increasing the temperature at 100, 125, 150, 175 and 200 °C and maintained at each step for 1 h. The thicknesses of the polymer films are the following: 40 µm for SPIN25, 30 µm for SPIN50, 30 µm for SPIN75 and 20 µm for SPIN100.

### 2.4. Laser Exposure

**Figure 1:**
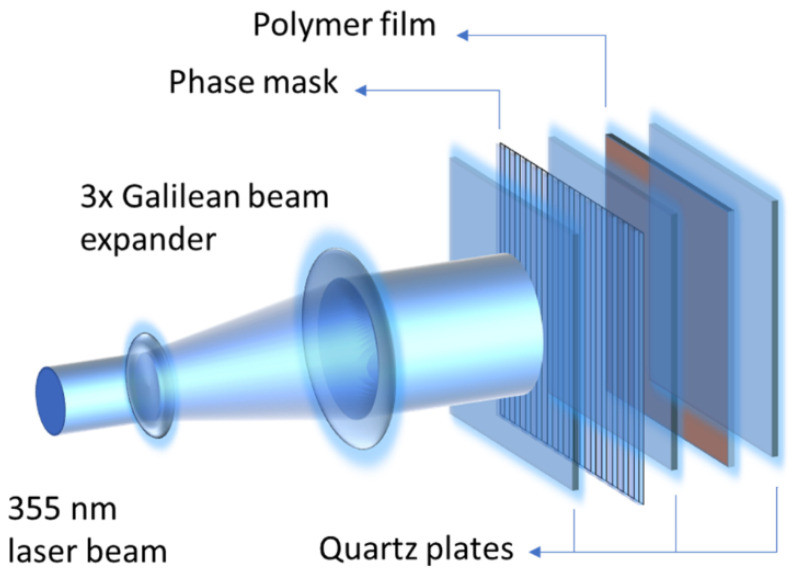
Laser irradiation sketch used for permanent relief grating generation on sample surface.

In Figure 1 is presented a sketch of the experimental arrangement used for the generation permanent relief grating on azo–copolyimide films. The third harmonic (355 nm) of a pulsed Nd:YAG laser (Brilliant, Quantel; 6 mm in diameter, 10 ns pulse length) directed through a 3× Galilean expander was used to illuminate a phase mask with 1000 grooves/mm (Edmund Optics Ltd., York, UK). In order to provide a laser irradiation spot with smooth energy distribution, the Galilean expander was interposed between two iris diaphragms. An iris with a 5 mm aperture was employed in the first step to pick the core region from the original Gaussian laser beam. This first cropping of the beam ensures clean edges of energy and, at the same time, avoids the residual corresponding to the second harmonic from reaching the irradiation area of the sample. The selected laser beam is then passed through the Galilean expander, from which a 10 mm central region is selected using a second iris diaphragm.

The generated near-field interference pattern was used for the fabrication of permanent relief grating with the same periodicity as that of the phase mask on PI-based supramolecular systems. The irradiation sequences were performed in air for different azo-copolyimide compositions while keeping constant the laser fluence (45 mJ/cm^2^), total delivered laser shots (300 laser pulses) and pulse repetition frequency (10 Hz). The attenuation factor of the assembly composed of the phase mask sandwiched between the two quartz plates was considered in order to estimate the laser fluence incident on the polymer sample. The laser-irradiated films have the following labels: SPIN25-L, SPIN50-L, SPIN75-L and SPIN100-L.

### 2.5. Characterization

Molecular modeling was undertaken with the help of HyperChem software. The molecular mechanics’ calculations involving Polak–Ribiere algorithm (0.001 kcal/mol) were used. Parametric method number 3 (PM3) was useful for quantum estimation of the molecular structure. The optimization is attained at a minimum free energy.

The quantitative structure–activity relationships (QSAR) properties could be employed to link molecular structure with chemical and physical properties. Molecular dynamics (MD) was useful for extraction of information on the van der Waals and hydrogen bonding interactions and the energetic and geometric quantities using Newtonian equations of motions derived from constituent atoms of the systems. Based on this, total energy (Etot), potential energy (Epot), and kinetic energy (Ekin) were evaluated.

The infrared spectroscopy (FTIR) experiments were carried out on FT-IR Bruker Vertex 70 instrument.

Differential scanning calorimetry (DSC) was experimented on a Mettler Toledo DSC 1 (Mettler Toledo, Greifensee, Switzerland) instrument. The polymer specimens were incorporated in some aluminum pans and subjected to a heating rate of 10 °C min^−1^ (under N_2_ medium).

Optical microscopy of the laser-exposed films was performed on a ADL 601 P Bresser device (Bresser, Rhede, Germany) in ambient temperature.

Illuminance and colorimetry studies were undertaken on CL-70F device (Konica Minolta, INC., Tokyo, Japan).

Refractometry and birefringence analyses were undertaken on DR-M4 (Atago Co., Ltd., Saitama, Japan) instrument under variable wavelength.

## 3. Results and Discussion

The new PI-based supramolecular systems were designed using the guest–host approach, and the employed strategy here was to emphasize the effect of the azochromophore content on the UV-induced properties. The samples were examined from a structural, thermal, morphological and optical point of view to analyze their utility as recording media for optical storage applications.

### 3.1. Molecular Modeling

**Figure 2:**
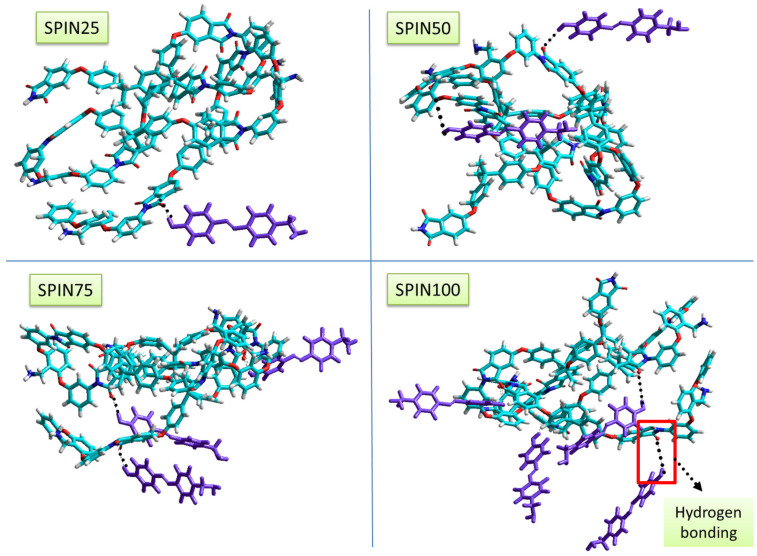
Geometry optimization (at minimal energy) of each PI-based supramolecular system (tube shape) containing various amounts of AzoCN. The PI (4 structural units) and AzoCN interact via hydrogen bonding. Colors: red is oxygen, cyan is carbon, withe is hydrogen, blue is nitrogen, and violet is AzoCN chromophore.

The performed simulations allowed the visualization of the geometrical conformation of the prepared PI-based supramolecular systems containing distinct amounts of azochromophore. Figure 2 presents the resulting simulation images displaying the full geometry optimization of the PI/AzoCN systems achieved for minimal energy in a vacuum. It can be observed that the synthetized cyano-containing PI is able to interact with AzoCN via hydrogen bonding, as supported by FTIR measurements.

**Figure 3:**
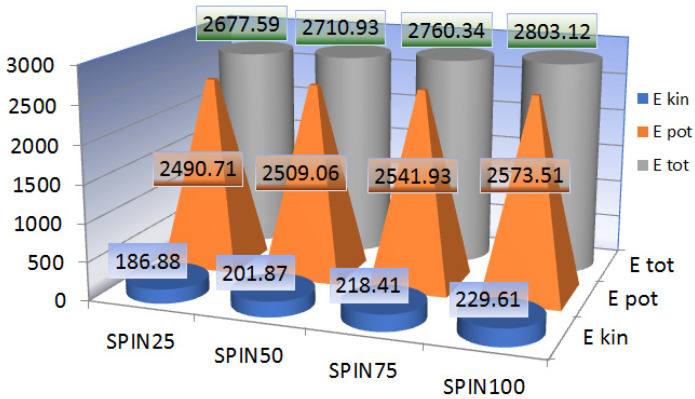
Total energy (Etot), potential energy (Epot) and kinetic energy (Ekin) values computed for the studied PI-based supramolecular systems.

QSAR enables the estimation of certain properties [37], such as the van der Waals surface-bounded molecular volume (Vw) and polarizability (P_0_). The results for these parameters are summarized in Table 2. The Vw is related to the van der Waals radius, so it denotes the volume of the molecular system. As shown in the computations, the magnitude of Vw increases as the content of the azochromophore is enhanced. The polarizability of the samples is also augmented by the incorporation of more AzoCN into the system. The MD simulations of the investigated structures are useful for extracting information on their energetic properties [38]. Figure 3 illustrates the variation of the potential energy (Epot) and kinetic energy (Ekin) when the molar ratio between PI and azomonomer was varied. As can be seen, the kinetic and potential energies increase as the percentage of AzoCN in the samples is enhanced.

### 3.2. FTIR Investigation

**Figure 4:**
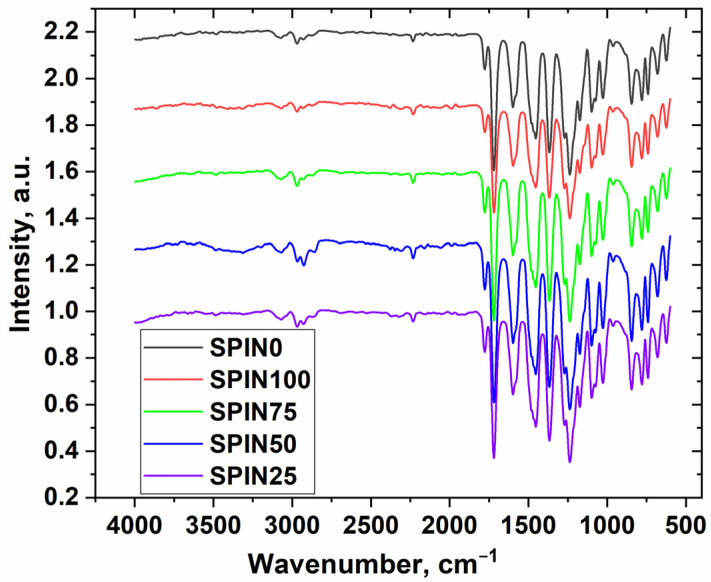
FTIR spectra of the pristine PI film, SPIN0 and PI-based supramolecular films SPIN25, SPIN50, SPIN75 and SPIN100.

Figure 4 depicts the FTIR spectra of the pristine PI film and the corresponding supramolecular structures. The characteristic absorption band of pristine PI films is present in all the analyzed compounds. The absorption peaks at 1780 and 1720 cm^−1^ which correspond to the C=O asymmetrical and symmetrical stretching of the PI and the absorption peaks at 1370 (imide III: C–N stretching vibration) and 720 cm^−1^ (imide IV: bending vibration of cyclic C=O) are also present. Other chemical groups were assigned as follows: aromatic –C–H linkages due to the absorption bands at 3066–3061 cm^−1^, the presence of CH_3_ groups at 2968–2853 cm^−1^ and aromatic ether due to the absorption bands at 1208–1134 cm^−1^. At the same time, the presence of the CN group in all the compounds is evidenced by the absorption characteristic band at 2230 cm^−1^. Azo-supramolecular polymer films showed the same characteristic absorption bands, such as pristine polymer film SPIN0, and some specific differences. The broad absorption band around 3350 cm^−1^ presented a slightly higher intensity, and the azo group, –N=N–, is overlapping with C=C of the aromatic rings at 1600 cm^−1^.

**Figure 5:**
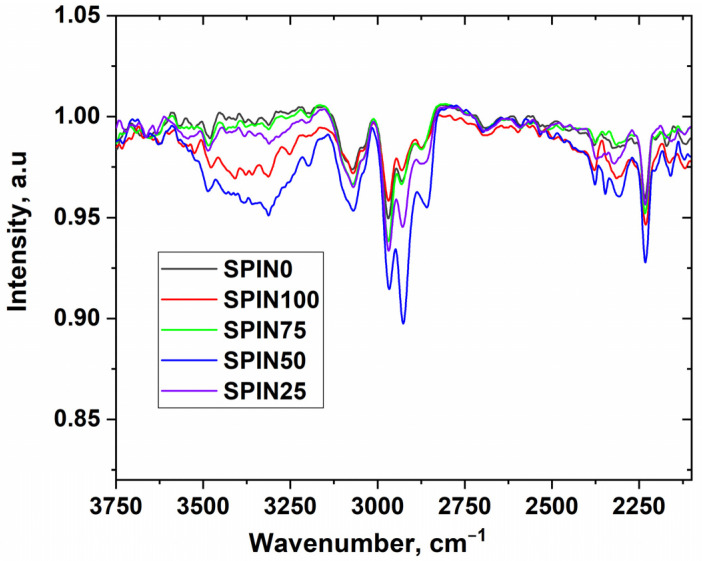
FTIR spectra of the analyzed compounds in the range of 3750–2100 cm^−1^.

For comparison, all the FTIR spectra are shown in Figure 5 in the range of 3750–2100 cm^−1^. As can be easily noted, the azo-PI supramolecular films (SPIN25, SPIN50, SPIN75 and SPIN100) present broad absorption bands around 3350 cm^−1^, which can be considered to be due to the formation of H-bonds between the carbonyl group in the imide rings and the hydrogen donor of the hydroxylic unit of the chromophore. Thus, the more intense absorption bands in the IR spectrum of the supramolecular azo-PI films situated in the range of 3600–3150 cm^−1^ are characteristic for H-bonds. This domain includes absorption bands of the valence vibrations of free or bounded aromatic hydroxyl groups and hydrogen bonds between the carbonyl in the imide rings and OH group from the chromophore [29]. Supramolecular azo-PI films exhibit the broad absorption band at around 3350 cm^−1^, probably due to the intermolecular H-bonded OH group. It is a clear evidence of the existence of the hydrogen bonds in the supramolecular azo-PI films (SPIN25, SPIN50, SPIN75 and SPIN100), as can be seen in the FTIR spectra in the range of 3600–3150 cm^−1^, but it is not possible to distinguish the difference between the distinct quantities of the AzoCN (Figure 5).

### 3.3. Thermal Properties

The thermal characteristics are essential for the utilization of these polymer materials in optical storage. The DSC experiments allowed the evaluation of the glass transition temperature (Tg) of each sample (see Supplementary Material, Figures S1–S5). The flexibility of the main chain is enhanced by the presence of the H_3_C–C–CH_3_ and –O– bridges that affect the internal rotation barrier and, implicitly, the magnitude of the Tg. According to Table 3, the inserted amount of azomonomer introduced in the supramolecular systems is slightly influencing the intermolecular interactions, and this is reflected in the sample thermal behavior. Hence, a higher quantity of the azochromophore is diminishing the interactions among the chains features, and through this, the Tg is slowly reduced with regard to the sample without azo dye (Figure S1). It is known that a bigger glass transition temperature is indicative of a higher chain rigidity, which, in turn, is linked to a larger residual birefringence [39]. However, in such situations, the maximum generated birefringence (by various external factors) is lower, presuming a constant chromophore concentration. Thus, the reduction in Tg by the presence of more AzoCN is beneficial for inducing a larger birefringence to the PI-based supramolecular systems. In any case, the Tg values are above 160 °C, which makes the examined PI-based supramolecular systems suitable for the optical storage uses. Moreover, based on the experimentally registered Tg of the samples, it is possible to extract information on the volumetric coefficient of thermal expansion (noted here as α_v_), as described in the work of Bicerano [40]. The obtained values are listed in Table 3, where it can be noted that the value of this parameter slightly changes upon enhancing the amount of the azo derivative in the sample. Thus, the small increase in α_v_ reveals a propensity for variation in the size of the sample at temperatures reaching the vitrification point.

### 3.4. Optical Microscopy

**Figure 6:**
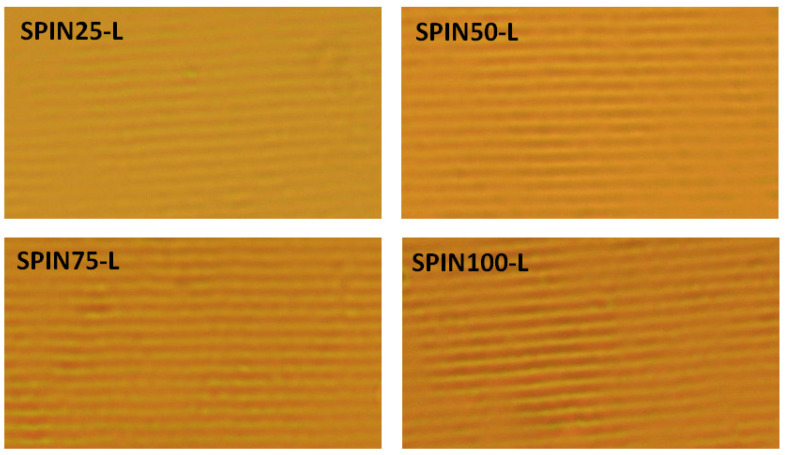
Optical microscopy pictures of the laser-exposed PI-based supramolecular systems.

The morphology of the laser-treated films was studied using optical microscopy. As seen in Figure 6, all samples display a wavy-like topography. This is caused by the presence of a photoactive component in the PI-based supramolecular systems, which enables the morphology under UV exposure to be controlled. The utilization of polarized light determines the selective excitation of the AzoCN and parallel disposition of its dipole to the direction of the radiation polarization. After stopping the irradiation procedure, some of the azo-based units tend towards molecular relaxation; however, a large amount of molecules keep their light-triggered orientation [41]. The observed periodic structures in azo-derived materials under UV laser treatment are known in the literature as surface relief gratings (SRGs) [29]. They are produced by a complex mechanism which combines radiation-driven mass transport and material fluidity [29,42]. In the case of the investigated samples, it is remarked that the addition of more AzoCN in the system makes the waves to be more clearly contoured. Thus, a greater surface anisotropy is favored by the higher amount of azochromophore. The mechanism of this phenomenon might be the following: a larger amount of azo dye in the system enhances the sensitivity to the action of polarized radiation. In this way, there are more AzoCN molecules that can be orientated via laser exposure, so that a larger part of the obtained material is aligned along the direction of the radiation polarization. Hence, the sample gains a larger surface anisotropy. The photogenerated anisotropy of morphology is expected to be reflected in the optical performance of the samples, namely, in gaining birefringence. The combination of bulk birefringence and the SRG pattern are useful for attaining recording layers with a good ability to store information.

### 3.5. Optical Properties

#### 3.5.1. Illuminance and Color Properties

**Figure 7:**
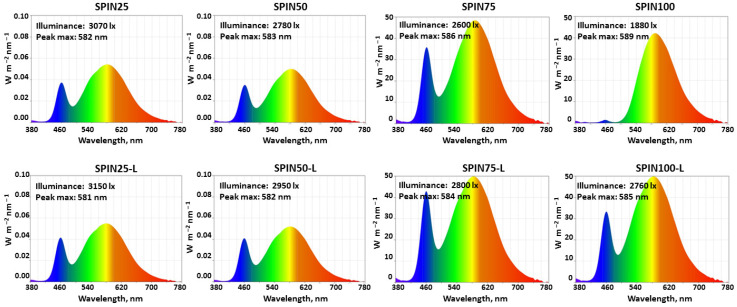
Spectral distribution of the visible radiations emitted by the LED recorded in the presence of the PI-based supramolecular systems before and after laser exposure.

The variation of the azo-derivative content in the PI systems produces changes in the appearance of the prepared films. To monitor this, tests of the illuminance and color features of the samples (under a LED light source) were performed. Figure 7 illustrates the spectral distribution of the visible radiations emitted by the LED, which are changed due to the presence of each PI/AzoCN system. The peak max parameter reveals the biggest wavelength optically emitted from the light source in the presence of the samples [43]. It can be observed that the peak max slightly increases with the addition of the azochromophore. In addition, the magnitude of the recorded illuminance is gradually reduced upon more azo dye incorporation. After laser irradiation, the illuminance values are enhanced, while the peak max values are slightly reduced with regard to the non-irradiated films. This might be due to the surface structuring which diminishes the light reflection into the incident medium and favors its propagation through the sample optical medium.

**Figure 8:**
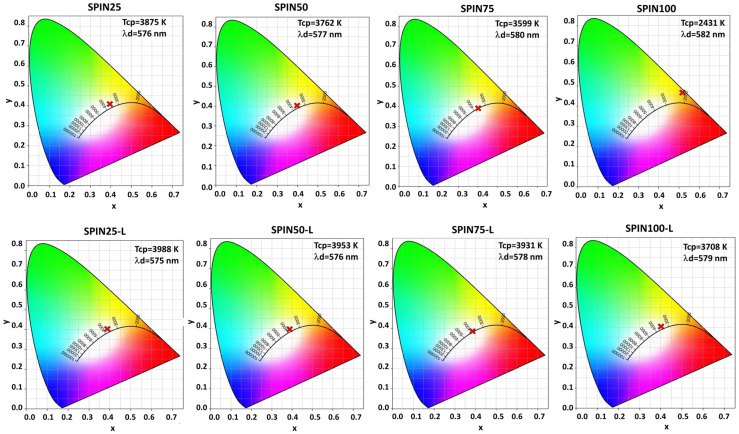
Chromaticity diagram of the light emitted by the LED recorded in the presence of the PI-based supramolecular systems before and after laser exposure.

The colorimetry experiments proved that both PI dyeing with AzoCN and UV laser treatment are generating effects that can be monitored using this technique. Figure 8 displays the chromaticity diagram of the light emitted by the LED recorded in the presence of the PI-based supramolecular systems before and after laser exposure. The correlated color temperature (denoted as Tcp) indicates the color appearance of the radiation released by a light source, linking its color to that of a reference source (usually a black-body radiator) at a similar temperature [43]. In the absence of the samples, the Tcp of the LED source is 6916 K. When placing the samples onto the measuring device, the Tcp corresponding to the light coming from the LED changes. By increasing the amount of AzoCN in the system, the Tcp is reduced for both the pristine and irradiated films. In Figure 8, there are also presented the data of the dominant light wavelength (λ_d_), which describes the color shade noticed by the human eye [43]. The incorporation of a larger amount of azo dye in the PI-based samples seems to enhance the magnitude of λ_d_. This shift towards higher wavelengths means that the samples are perceived to display a yellowing effect upon doping with the selected azochromophore. For the laser-exposed films, the same tendency is remarked, but the values of the λ_d_ parameter are slightly smaller with regard to their non-irradiated counterpart. Thus, the changes in the color shade of the samples, is a good indicative of proper dyeing with the chosen light-sensitive compound.

#### 3.5.2. Light Dispersion and Related Parameters

**Figure 9:**
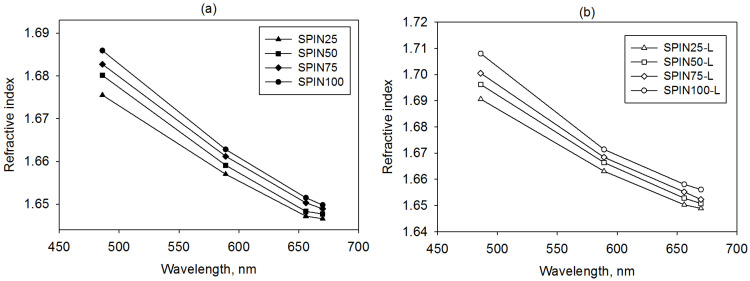
Light dispersion plots corresponding to (**a**) non-irradiated and (**b**) laser-exposed PI-based supramolecular systems.

The speed of the optical radiations moving through the air/sample media can be quantified using refractive index (n) experiments. As noted in Figure 9, this parameter can suffer relevant changes upon introduction of the azo dye in the system. This is because the polar AzoCN molecules are increasing the overall polarizability of the system; hence, the magnitude of n is augmented, meaning that the visible radiations are slowed down after gradually doping the samples. Such an influence of dye on the refractive properties of other type of polymers was reported by Abdelrazek [44]. Moreover, the manner in which the refractive index ranges with wavelength is affected by the film composition, namely, the values of n are more increased towards wavelengths below 650 nm. This accentuates the power of the optical radiation to split into constituent colors when refracted by the sample. Upon UV laser treatment, the prepared films display a higher refractive index in comparison to the pristine ones. This result is supported by Cimrova [45], who also observed an increase in n after the laser exposure of azobenzene-containing copolymers. For the analyzed laser-exposed polymer sheets, the differences between n values at the lowest wavelengths are a little bigger (with regard to the pristine films), so the dispersion properties are slightly enhanced. The influence of the dye content in PI materials on the light dispersion properties has not been emphasized in the literature yet, as far as we know.

The registered light dispersion properties of the PI-based supramolecular systems enable the calculation of important dispersion-related parameters. Through utilization of the Wemple and DiDomenico (WDD) theory [46], it is possible to analyze the spectral dependence of the refractive index under the band gap energy region (interband absorption edge). Accordingly, the refractive index can be linked to photon energy, as depicted in the below relationship (1):

(1) $$n^{2}=1+\frac{{E_{d}E}_{0}}{E_{0}^{2}-E^{2}},$$

where E is the photon energy, E_d_ is the dispersion energy, and E_0_ is the single oscillator energy.

**Figure 10:**
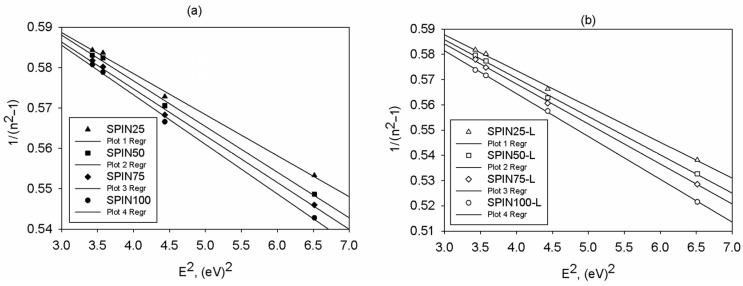
(n^2^ − 1)^−1^ vs. E^2^ plots of (**a**) non-irradiated and (**b**) laser-exposed PI-based supramolecular systems.

The parameters E_d_ and E_0_ can be extracted from the plot of (n^2^ − 1)^−1^ versus the square of the photon energy. Figure 10 illustrates these graphs for the examined films under the form of straight lines. The data of E_d_ and E_0_ are summarized in Table 4.

The data of refractive index dispersion at energies under the interband absorption edge can be attributed to the fundamental electronic excitation spectrum. The E_0_ parameter offers relevant information on the band structure of the polymeric material, while E_d_ is connected to the mean potency of interband photosensitive transitions, including structural variations [47]. The results reveal that the introduction of AzoCN in the system determines the diminishment of the strength of the interband transition and the average excitation for electronic transitions (quantified by E_0_). This aspect is further accentuated by the laser treatment of the samples. The changes in strength of the interband optical transitions reveal the dye-induced modification of the microstructural properties, which are affecting the electron/hole excitations in the energy-dependent fundamental electronic excitation spectrum. In addition to this, as was suggested by Tanaka [48], an estimative value of the optical band gap (E_g_) can be obtained from the oscillator energy. The attained E_g_ values for the samples before and after UV irradiation are shown in Table 4. The increasing amount of azochromophore has the effect of reducing the band gap of the studied systems. This is consistent with the report of Abdelrazek [44] for sulfone-based polymers doped with another azo dye. It seems that the presence of AzoCN favors the occurrence of localized states close to conduction or valance bands owing to the vacancies, defects and interstitials. This strongly influences the optical band gap energy but does not display a pronounced effect on the oscillator energy. Aside from the azo-component addition, laser exposure enhances the appearance of localized states and lowers the forbidden gap with regard to the non-irradiated counterpart samples.

Furthermore, the WDD approach is useful for extracting additional optical parameters, reflecting the linear and non-linear optical properties of the studied systems, such as the zero frequency refractive index (n_0_), linear optical susceptibility (ζ(1)), third-order optical susceptibility (ζ (3)) and the nonlinear refractive index (n_NL_), as shown in Equations (2)–(4):

(2) $$n_{0}={\left(1+E_{d}/E_{0}\right)}^{1/2},$$

(3) $$\zeta (1)=\frac{1}{4\pi }(E_{d}/E_{0}),$$

(4) $$\zeta \left(3\right)=6.82\times 10^{-15}\times {\left(E_{d}/E_{0}\right)}^{4},$$

(5) $$n_{NL}=\frac{12\pi \zeta (3)}{n_{0}}.$$

The calculated values of the aforementioned parameters are listed in Table 4. The static refractive index shows a decrease when more AzoCN is present in the samples. In addition, a resembling tendency is observed for the non-linear optical parameters. Laser treatment further reduces the values of these optical constants when compared to the pristine films. It appears that the non-linear optical performance of the prepared materials can be controlled via the composition of the films and exposure to polarized light.

The studied PI-based supramolecular systems are also able to respond as a result of the charge carrier motions in the presence of optical radiations. This aspect is quantified by optical conductivity (σ_0_), which is a function of the refractive index and absorption coefficient, as indicated in Equation (6):

(6) $$\sigma_{0}=\frac{\alpha nc}{4\pi },$$

where α is the absorption coefficient, and c denotes the light velocity in a vacuum.

**Figure 11:**
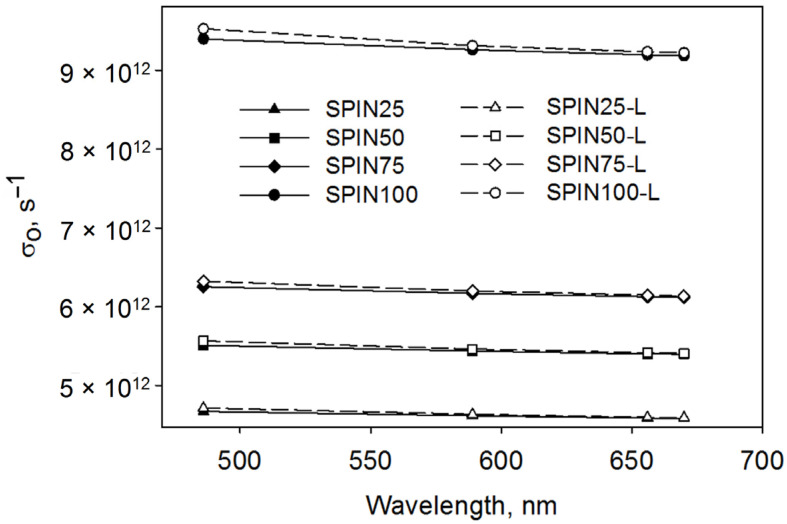
Optical conductivity of the PI-based supramolecular systems before and after laser exposure.

Figure 11 displays the wavelength variation of the optical conductivity of the analyzed films. At a constant azo dye content, the data are revealing a slight decrease of σ_0_ towards lower photon energies. When changing the system composition by adding more AzoCN, the optical conductivity is augmented. Laser irradiation of the films determines a slight increase in σ_0_ at smaller wavelengths with regard to the initial samples. The increase in the samples’ conductivity at optical frequencies, especially after exposure, is beneficial for optical storage purposes as, in this way, the recording medium interacts better with polarized radiations from UV and a visible range, facilitating the information writing process.

#### 3.5.3. Birefringence

**Figure 12:**
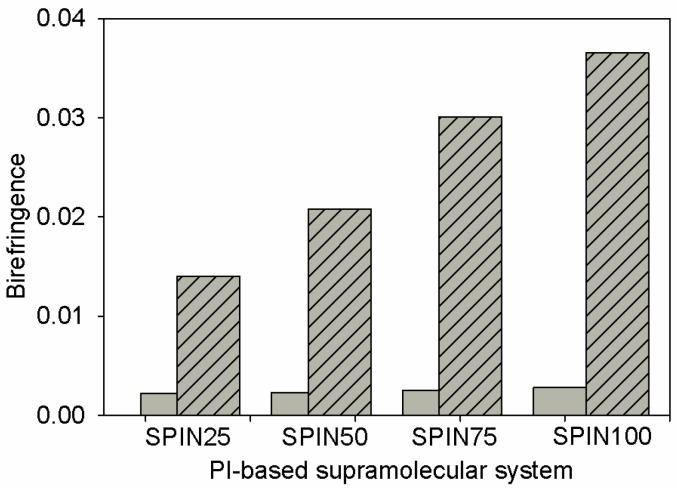
Birefringence of the PI-based supramolecular systems with AzoCN content before and after laser exposure. The bar containing a pattern represents the laser-exposed samples.

Birefringence appears to be the key optical parameter of a material that is able to record data in an optical storage device [41]. In the case of writeable media, the ‘bit’ occurs in the recording layer that is exposed to a laser beam of elevated power during several tens of seconds. Therefore, the writing mechanism in an optical storage system involves an optically generated birefringence. Figure 12 presents the birefringence data of the samples. As noticed, the pristine samples display an inherent birefringence, which is gradually increased by the incorporation of AzoCN. Birefringence was also reported in other polymer/azo dye systems [49]. The power from the laser pulse produces certain modifications in the PI/AzoCN recording layer that are reflected in a larger birefringence (coarse bars from Figure 12). The orientation of the PI-based films is more pronounced as the azo- dye content is larger. In this case, there are more photosensitive molecules in the samples that can suffer light-triggered orientation, hence higher birefringence. The induction of such stable birefringence denotes the good ability of the highly AzoCN-doped samples to store information upon laser irradiation, as demanded by optical storage applications.

## 4. Conclusions

In this work, the potential use of polyimide-based films as a recording medium for optical storage devices was analyzed. For the structural design of the samples, the host–guest approach was employed for a paired PI precursor and azo dye (with the following ratios: 1:0.25, 1:0.5, 1:0.75 and 1:1), which, after thermal treatment, led to supramolecular systems. The molecular modeling and infrared spectroscopy emphasized that the PI and azochromophore interact via hydrogen bonding, as supported by the broad band at 3350 cm^−1^. The thermal analysis showed a reduction in the vitrification temperature from 190.84 °C for the sample without azo dye to 163.91 °C for the SPIN100 film. This is shown to be beneficial for inducing birefringence by means of laser irradiation. Optical microscopy illustrated that the laser-exposed films are able to develop a regular pattern, which along with photo-induced birefringence, is useful for achieving recording layers with a higher ability to store information. The colorimetry tests revealed changes in the sample aspect produced by both the addition of AzoCN and laser irradiation; namely, the correlated color temperature decreases, while the dominant wavelength shifts towards higher values upon dyeing the films and UV light treatment. The incorporation of polar azochromophore into the system increases the refractive index from 1.657 (SPIN25) to 1.663 (SPIN100) at 589 nm, while the optical band gap decreases from 3.92 (SPIN25) to 3.56 eV (SPIN100) for the unexposed samples. After laser treatment, n is further increased (1.664–1.671 at 589 nm), and Eg is additionally diminished (3.33–3.12 eV). The values of the non–linear optical parameters are slightly reduced upon sample doping with AzoCN and laser treatment. At the same time, the birefringence is enhanced from 0.0012 (SPIN25) to 0.0028 (SPIN100) upon AzoCN insertion, and this is further augmented by laser irradiation as follows: 0.014 (SPIN25-L), 0.0208 (SPIN50-L), 0.0301 (SPIN75-L) and 0.0365 (SPIN100-L). These results recommend the use of the studied materials as recording media in optical storage applications.

## Figures and Tables

**Table 1:** Quantities of polyamidic acid and azo monomer used in the PI-based supramolecular systems.

Sample Code	Polyamidic Acid Mol (g)	Azo Monomer (Azo CN) Mol (g)
SPIN25	0.1806 × 10^−4^ (0.151)	0.045 × 10^−4^ (0.01007)
SPIN50	0.1806 × 10^−4^ (0.151)	0.09 × 10^−4^ (0.020137)
SPIN75	0.1806 × 10^−4^ (0.151)	0.135 × 10^−4^ (0.0302)
SPIN100	0.1806 × 10^−4^ (0.151)	0.18 × 10^−4^ (0.04027)

**Table 2:** Van der Waals volume and polarizability computed for the PI-based supramolecular systems.

Sample Code	Vw (Å^3^)	P_0_ (Å^3^)
SPIN25	2923.65	375.15
SPIN50	3147.13	402.11
SPIN75	3358.33	426.95
SPIN100	3700.15	452.36

**Table 3:** Thermal properties of the analyzed PI-based supramolecular systems: glass transition temperature (Tg) and thermal expansion (α_v_).

Sample Code	Tg (°C)	α_v_ (1/°C)
SPIN0	190.84	4.75 × 10^−4^
SPIN25	176.60	5.08 × 10^−4^
SPIN50	171.30	5.21 × 10^−4^
SPIN75	164.68	5.38 × 10^−4^
SPIN100	163.91	5.41 × 10^−4^

**Table 4:** Light dispersion parameters for the PI-based supramolecular systems.

Sample Code	E_d_ (eV)	E_0_ (eV)	E_g_ (eV)	n_0_	ζ (1) (e.s.u.)	$\zeta \;(3)\times 10^{14}$ (e.s.u.)	n_NL_, $\times 10^{13}$ (e.s.u.)
SPIN25	12.66	7.83	3.92	2.272	0.1287	4.6609	7.7314
SPIN50	12.09	7.52	3.76	2.268	0.1280	4.5564	7.5700
SPIN75	11.69	7.31	3.65	2.265	0.1273	4.4684	7.4216
SPIN100	11.38	7.12	3.56	2.264	0.1272	4.4508	7.4066
SPIN25-L	10.57	6.65	3.33	2.261	0.1266	4.3531	7.2553
SPIN50-L	10.21	6.45	3.23	2.258	0.1260	4.2820	7.1451
SPIN75-L	10.08	6.38	3.19	2.257	0.1258	4.2496	7.0947
SPIN100-L	9.85	6.25	3.12	2.256	0.1255	4.2108	7.0344

## Data Availability

Not applicable.

## Supplementary Materials

The following supporting information can be downloaded at: https://www.mdpi.com/2073-4360/15/4/1056/s1, Figures S1–S5: DSC curve of SPIN0, SPIN25, SPIN50, SPIN75 and SPIN100 samples.
