# Supplementary material for: Polyimide-Derived Supramolecular Systems Containing Various Amounts of Azochromophore for Optical Storage Uses
Source: Polymers (Basel). 2023 Feb 20;15(4):1056. doi: 10.3390/polym15041056 (PMC10004395; doi:10.3390/polym15041056)
Supplement: Supplementary file 1 [file polymers-15-01056-s001.zip › polymers-2221467-supplementary.pdf]

Polyimide-derived supramolecular systems containing various amounts of an azo-derivative for optical storage uses

Andreea Irina Barzic[1], Ion Sava[1], Raluca Marinica Albu[1], Cristian Ursu[1], Gabriela Lisa[2] and Iuliana Stoica[1,*]

[1] "Petru Poni" Institute of Macromolecular Chemistry, 41A Grigore Ghica Voda Alley, 700487 Iasi, Romania

[2] Faculty of Chemical Engineering and Environmental Protection "Cristofor Simionescu", "Gheorghe Asachi" Technical University of Iasi-Romania, 73 Prof. dr. doc. D. Mangeron Street, 700050 Iasi, Romania

* Correspondence: stoica_iuliana@icmpp.ro (I.S.)

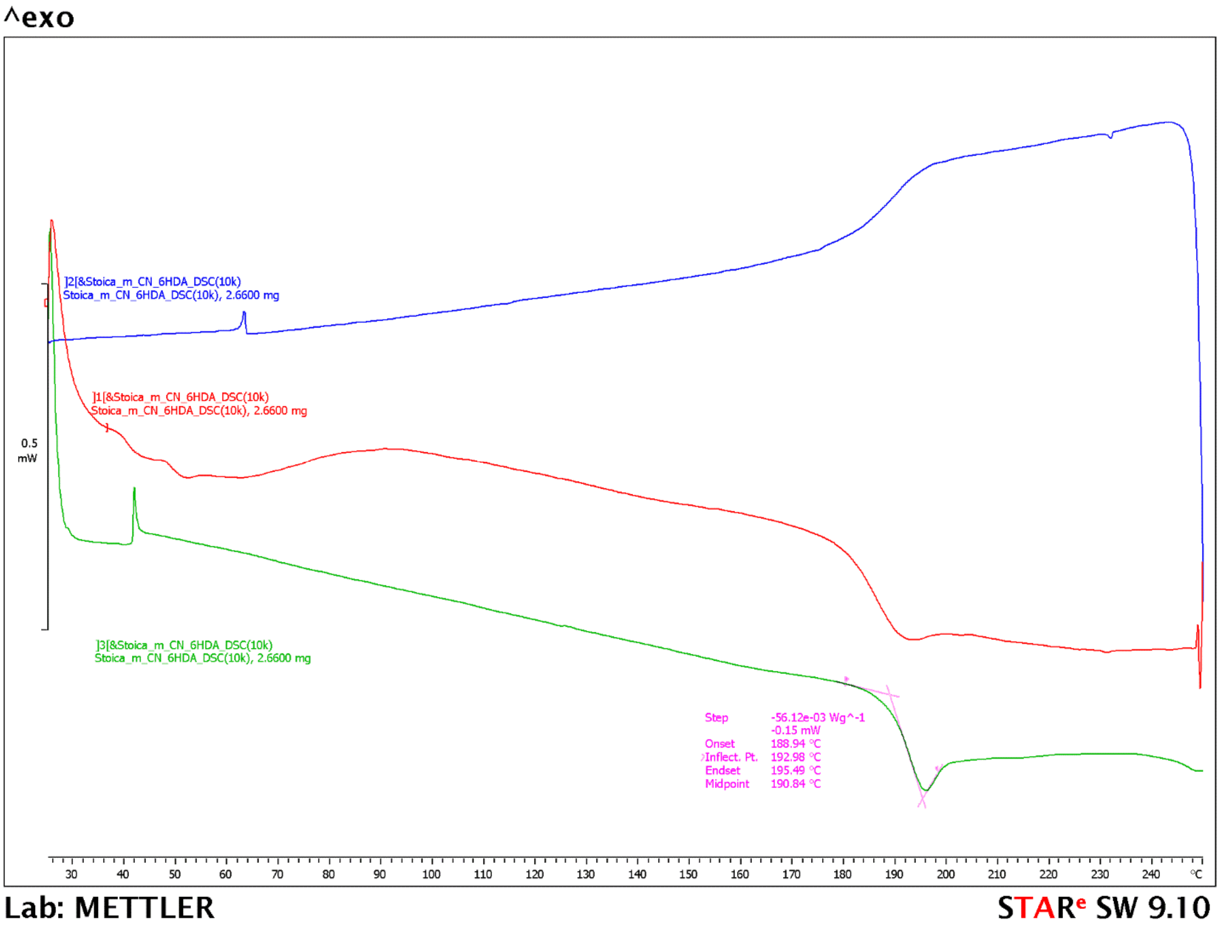

Figure S1. DSC curve of SPIN0 sample.

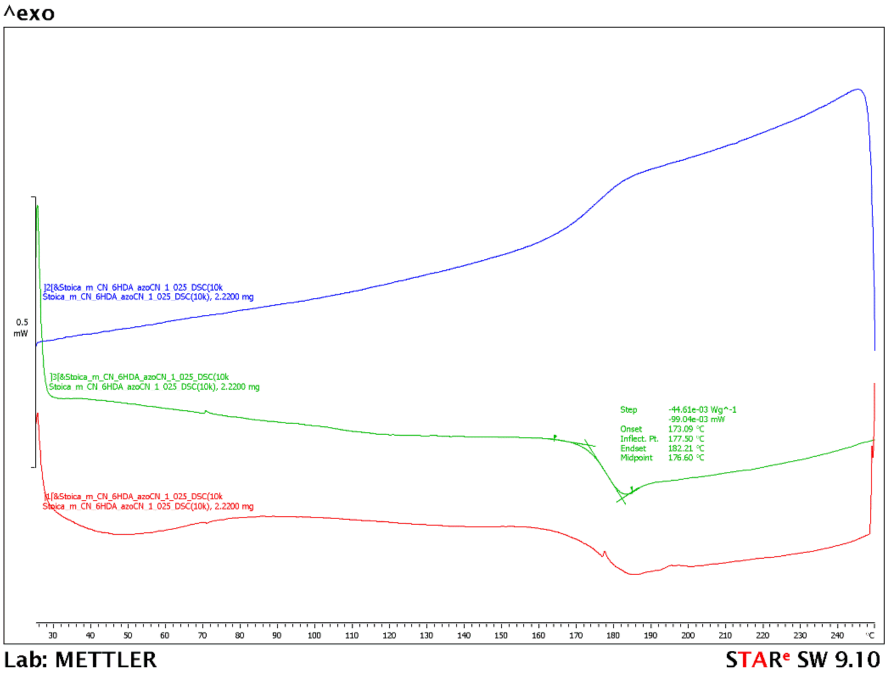

Figure S2. DSC curve of SPIN25 sample.

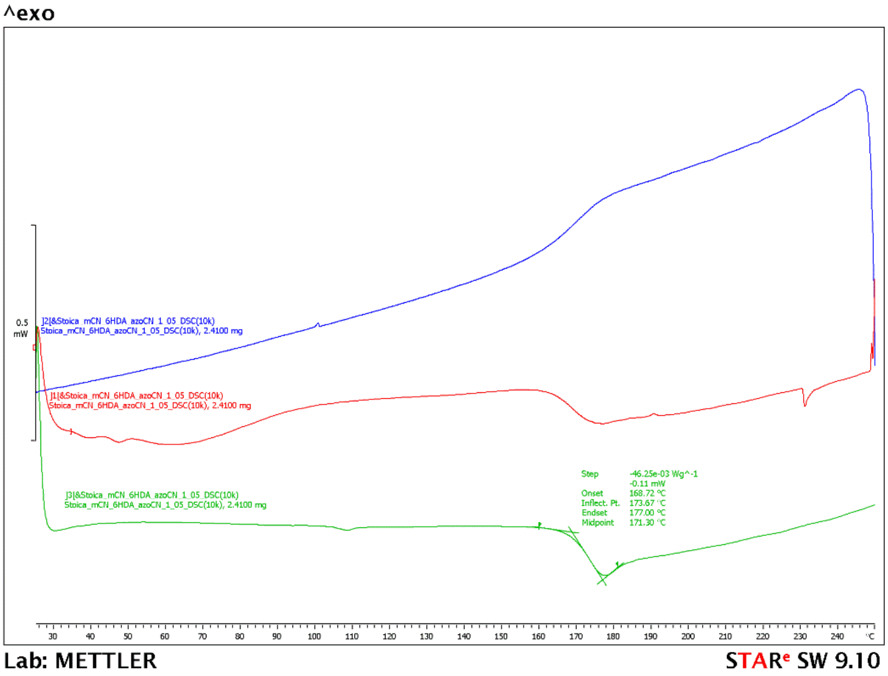

Figure S3. DSC curve of SPIN50 sample.

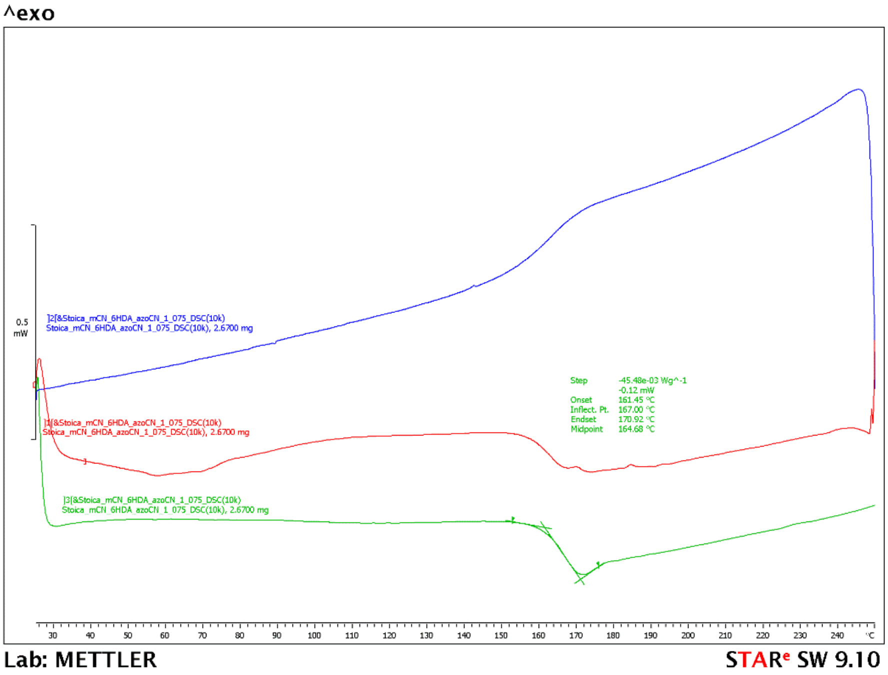

Figure S4. DSC curve of SPIN75 sample.

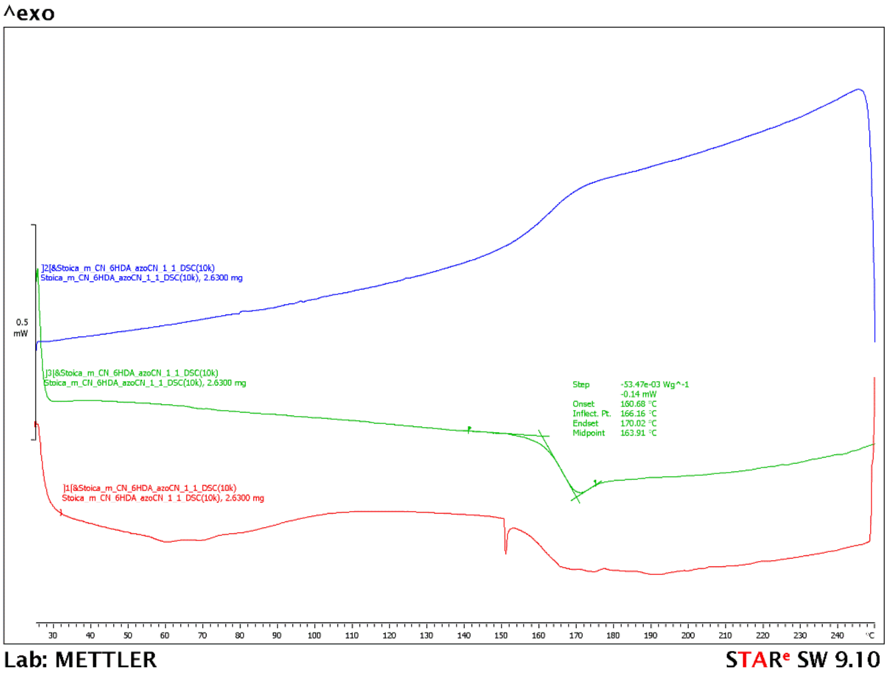

Figure S5. DSC curve of SPIN100 sample.
